# Feasibility of transitioning from APACHE II to SAPS III as prognostic model in a Brazilian general ICU

**DOI:** 10.1186/cc12626

**Published:** 2013-06-19

**Authors:** A Serpa Neto, MSC Cesar de Assunção, A Pardini, NS Pinheiro de Goes, E Silva

**Affiliations:** 1 Hospital Israelita Albert Einstein, Morumbi, São Paulo, SP, Brazil

## Introduction

Prognostic models reflect the population characteristics of the countries which they originate from. The development of the Acute Physiology and Chronic Health Evaluation II (APACHE II) system was based on a cohort of patients in the United States, and it has been used in many ICUs around the world. Newer score systems were developed as the Simplified Acute Physiology Score III (SAPS III) that was developed and validated in a multicenter and multinational cohort study. Predictive models should be customized to fit in the case-mix where they will be used because the outcomes in the original databases and period from which the models were derived may be different from the databases of ICUs using the models. In the present study, we performed the external validation of two predictive models and directly compared their performance in an independent population of mixed critically ill patients in Brazil. The aim is to assess the feasibility of transitioning from APACHE II to SAPS III.

## Methods

Data were retrospectively collected only for APACHE II during August 2011 and December 2011, and only for SAPS III during May 2012 and September 2012. From January 2012 to April 2012, during a period of calibration, the two scores were calculated in all patients admitted to the ICU and were collected for analysis. All ICU admissions were enrolled during the period analyzed. The exclusion criteria were: age <18 years, missing data, and not receiving ICU care. The calibration of the scores was tested using the Hosmer-Lemeshow goodness-of-fit procedure. The discriminative ability of the models was assessed using receiver operating characteristic (ROC) curves and respective areas under curves (AUC). The standardized mortality ratio (SMR) was calculated using the models by dividing the number of observed deaths by the number of expected deaths. Confidence intervals of the SMR were computed to test the model's uniformity-of-fit and were calculated using the proposed methods.

## Results

A total of 3,333 ICU admissions were enrolled until the end of September 2012. The Hosmer-Lemeshow goodness-of-fit statistics supported model fit of all models for in-ICU mortality with the exception of APACHE II in patients in the calibration database undergoing elective surgery. For in-hospital mortality there is a worse fit of APACHE II in clinical patients during the first period and of SAPS III in patients in the calibration database undergoing elective surgery. The calibration curves for APACHE II and SAPS III shows overestimation of the risk of death in all ranges of predicted mortality. Discrimination, as tested by the AUC, in general and clinical patients was best for SAPS III for in-ICU and in-hospital mortality. SMRs for the whole population were 0.27 (CI = 0.23 to 0.33) for APACHE II and 0.28 (CI = 0.22 to 0.36) for SAPS III. In the calibration database, the SMRs for APACHE II and SAPS III were 0.33 (CI = 0.22 -to 0.50) and 0.36 (CI = 0.25 to 0.55), respectively. For all models, the SMRs showed some variation across the spectrum of patients. The SMRs ranged from 0.24 to 0.46 for APACHE II, and 0.09 to 0.31 for SAPS III. In the calibration database, the SMRs ranged from 0.13 to 0.38 for APACHE II, and from 0.18 to 0.40 for SAPS III.

## Conclusion

The external validation of two widely used prognostic models showed good discrimination and good calibration when applied to the same independent population of Brazilian ICU patients. The transition from APACHE II to SAPS III in this Brazilian ICU was feasible and in some scenarios the SAPS III had even better performance than APACHE II. In conclusion, we showed in a cohort of Brazilian patients from a tertiary hospital that SAPS III is the best prognostic score, with the highest discrimination and calibration power. The transition from an older score (APACHE II) to a newer one (SAPS III) is feasible in this scenario.

